# Leptomeningeal Carcinomatosis During Dose-Reduced Osimertinib Therapy in Epidermal Growth Factor Receptor (EGFR) L858R-Mutated Lung Adenocarcinoma: A Case Report

**DOI:** 10.7759/cureus.107914

**Published:** 2026-04-28

**Authors:** Yu Tsukasa, Yukihiro Sugimoto, Takayuki Yamamoto, Ryota Aoki, Hirofumi Nakano

**Affiliations:** 1 Department of Respiratory Medicine, Fukuoka Seisyukai Hospital, Fukuoka, JPN

**Keywords:** egfr-mutant lung adenocarcinoma, egfr tyrosine kinase inhibitors, leptomeningeal carcinomatosis, non-small cell lung cancer, thrombocytopenia

## Abstract

Leptomeningeal carcinomatosis (LMC) remains a devastating complication of epidermal growth factor receptor (EGFR)-mutated non-small cell lung cancer despite the availability of central nervous system (CNS)-active EGFR tyrosine kinase inhibitors (TKIs). We report the case of a 77-year-old woman with EGFR L858R-mutated lung adenocarcinoma (cT1cN3M1c stage IVB) who initiated first-line osimertinib 80 mg/day. Severe hematologic toxicity required sequential dose reductions to 40 mg/day and subsequently 40 mg every other day. Although extracranial disease remained well controlled, the patient developed LMC three months after treatment initiation, accompanied by a rapid rise in carcinoembryonic antigen and sialyl Lewis X levels. Erlotinib 150 mg/day was introduced based on reports of dose‑dependent CNS efficacy, resulting in prompt improvement of neurological symptoms; however, grade 3 thrombocytopenia led to treatment interruption, followed by rapid neurological decline and death. This case illustrates that reduced‑dose EGFR TKI therapy, even with a CNS‑penetrant agent such as osimertinib, may provide insufficient cerebrospinal fluid drug exposure to prevent CNS progression. Clinicians should recognize that dose modification necessitated by adverse events may compromise CNS control, and vigilant monitoring for neurological symptoms is essential in patients receiving reduced‑dose EGFR TKIs.

## Introduction

Leptomeningeal carcinomatosis (LMC) is a rare but devastating complication of non‑small cell lung cancer and is associated with poor outcomes [1]. In patients with epidermal growth factor receptor (EGFR)‑mutated disease, EGFR tyrosine kinase inhibitors (TKIs) have improved survival and demonstrate meaningful central nervous system (CNS) activity [2].

Although dose reduction of EGFR TKIs is frequently required in real‑world practice due to hematologic or non‑hematologic toxicities, the impact of reduced dosing on CNS control remains poorly defined. In particular, evidence regarding CNS outcomes in patients receiving dose‑reduced osimertinib is very limited. Osimertinib, a third‑generation EGFR TKI with established CNS penetration, is generally effective against LMC, but its efficacy under dose‑reduced conditions has not been well characterized.

We encountered a patient with EGFR L858R‑mutated lung adenocarcinoma who developed LMC despite maintaining systemic disease control on reduced‑dose osimertinib. This case highlights the potential limitations of dose‑reduced EGFR TKI therapy in preventing CNS progression.

## Case presentation

A 77‑year‑old woman fell at home while carrying a meal tray and developed right thigh pain with difficulty moving, leading to emergency transport to our hospital on June 1. Pelvic radiography revealed a right femoral neck fracture and sclerotic changes of the femoral head, raising suspicion of bone metastasis. Whole‑body evaluation identified a mass in the right upper lobe on chest CT, and she was referred to our department for suspected primary lung cancer. On June 13, bronchoscopy was performed post surgical repair of the fracture, which confirmed lung adenocarcinoma. Staging revealed cT1cN3M1c stage IVB disease with bone, lymph node, and liver metastases, EGFR L858R mutation positivity, and programmed death-ligand 1 Tumor Proportion Score (PD‑L1 TPS) 1-24%. 

Her medical history included hypertension, colonic polyps, cystitis, and bilateral soleal vein thrombosis. She had no smoking history. and regular medications included propiverine hydrochloride 20 mg/day, edoxaban 30 mg/day, valsartan 10 mg/day, pregabalin 75 mg/day, rebamipide 200 mg/day, celecoxib 200 mg/day, tramadol hydrochloride 100 mg/day, and acetaminophen 1200 mg/day.

At admission, her height was 157.0 cm, weight 47.6 kg, temperature 36.2°C, blood pressure 104/66 mmHg, pulse 82 beats per minute, respiratory rate 14 breaths per minute, and oxygen saturation 98% on room air. Her performance status was 1. Auscultation revealed decreased breath sounds in the right upper and lower lung zones. Laboratory tests (Table 1) showed elevated alanine aminotransferase (ALT) (494 U/L) and lactate dehydrogenase (LDH) (525 U/L), mild anemia (Hb 10.6 g/dL), and markedly elevated tumor markers (carcinoembryonic antigen (CEA) 194.4 ng/mL, sialyl Lewis X (SLX) 364 U/mL).

**Table 1:** Laboratory findings at admission

Parameter	Patient Value	Reference Range
Hematology		
White blood cell count	5,750 /µL	3,300–9,000 /µL
Neutrophils	62.4 %	—
Lymphocytes	25.8 %	—
Monocytes	7.4 %	—
Eosinophils	3.6 %	—
Basophils	0.8 %	—
Hemoglobin	10.6 g/dL	11.5–15.0 g/dL
Platelet count	30.8 ×10⁴/µL	12.0–35.0 ×10⁴/µL
Biochemistry		
Total protein	6.2 g/dL	6.7–8.3 g/dL
Albumin	3.6 g/dL	4.0–5.0 g/dL
Total bilirubin	0.73 mg/dL	0.30–1.20 mg/dL
Aspartate aminotransferase (AST)	34 U/L	13–33 U/L
Alanine aminotransferase (ALT)	14 U/L	6–30 U/L
Alkaline phosphatase (ALP)	494 U/L	38–113 U/L
γ-glutamyl transpeptidase (γ-GTP)	43 U/L	10–47 U/L
Lactate dehydrogenase (LDH)	525 U/L	119–229 U/L
Blood urea nitrogen	21.2 mg/dL	8.0–22.0 mg/dL
Creatinine	0.72 mg/dL	0.40–0.70 mg/dL
C-reactive protein	0.38 mg/dL	<0.30 mg/dL
Electrolytes		
Sodium	140 mmol/L	138–146 mmol/L
Potassium	4.1 mmol/L	3.6–4.9 mmol/L
Chloride	103 mmol/L	99–109 mmol/L
Tumor markers		
Carcinoembryonic antigen (CEA)	408.9 ng/mL	<5.0 ng/mL
Sialyl Lewis X (SLX)	364 U/mL	<38.0 U/mL
Coagulation		
Prothrombin time (PT)	12.3 s	—
PT–international normalized ratio (PT-INR)	1.06	0.85–1.15
Activated partial thromboplastin time (APTT)	28.1 s	27.0–38.0 s

**Figure 1:**
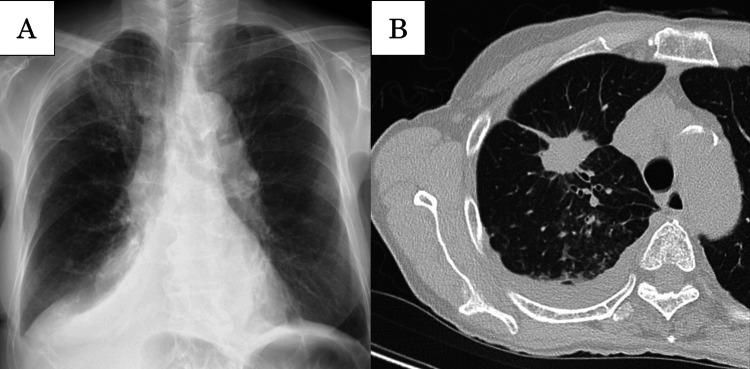
Chest radiography and computed tomography of the chest at admission. (A) Chest radiography demonstrated a ground‑glass opacity in the right upper lung zone, accompanied by a right pleural effusion. (B) Computed tomography of the chest revealed a 26‑mm nodule with irregular margins in the right upper lobe, together with a right pleural effusion.

Chest radiography demonstrated a ground‑glass opacity in the right upper lung zone and right pleural effusion (Figure 1A). Chest CT revealed a 26‑mm irregular nodule in the right upper lobe and right pleural effusion (Figure 1B). Additional findings included multiple enlarged mediastinal lymph nodes, faint low‑attenuation hepatic nodules, and ill‑defined sclerotic lesions in the thoracolumbar spine, sacrum, and ribs.

Although treatment for the right femoral neck fracture was initially prioritized, the primary lung lesion enlarged and the right pleural effusion increased over the first month of hospitalization, accompanied by the onset of dyspnea. Therefore, lung cancer treatment was initiated earlier than originally planned. Osimertinib 80 mg/day was started on July 3 (day 1) as first‑line therapy. By day 5, platelet counts decreased from 320,000/µL to 56,000/µL (grade 2), prompting treatment interruption. Platelets recovered spontaneously; however, hemoglobin declined from 9.2 g/dL to 6.8 g/dL (grade 3). Given the rapid hemoglobin decline, stable LDH, and concomitant use of anticoagulants and nonsteroidal anti-inflammatory drugs (NSAIDs), gastrointestinal bleeding was suspected, but upper endoscopy revealed no active bleeding. Mild gastritis was observed, and rabeprazole 10 mg/day was started for dyspepsia and anorexia. The anemia was attributed to osimertinib toxicity, and four units of red blood cells were transfused on day 16. Osimertinib was resumed at 40 mg/day on July 20 (day 18).

**Figure 2:**
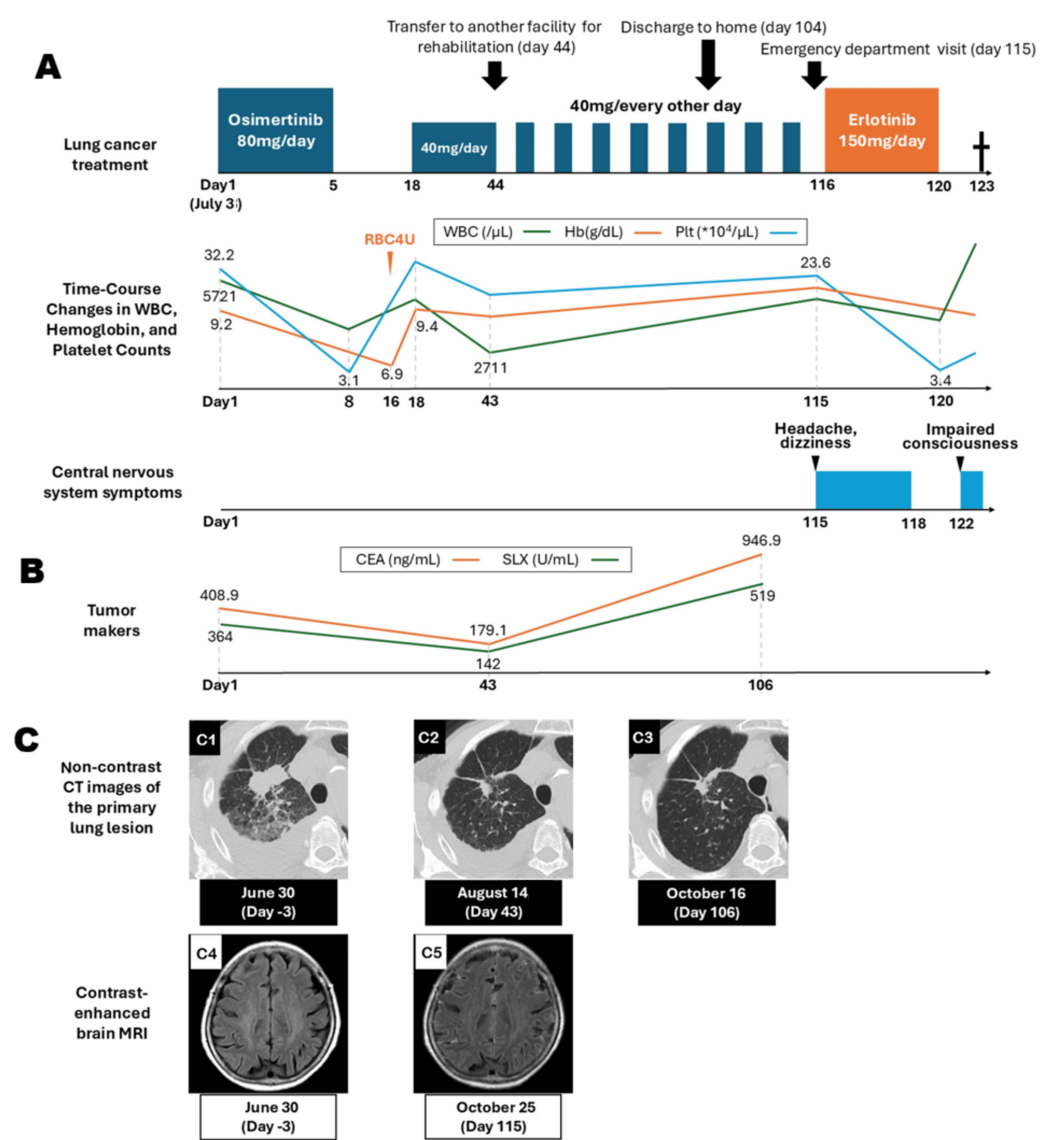
Clinical course during treatment (A) With osimertinib 80 mg/day, grade 3 cytopenia developed, requiring red blood cell transfusion and subsequent dose reductions to 40 mg/day and then 40 mg every other day. During second‑line erlotinib therapy, grade 3 thrombocytopenia recurred, leading to treatment interruption, after which the patient rapidly developed impaired consciousness and died. No central nervous system symptoms were noted early in the course, but headache and dizziness appeared on day 115 during reduced‑dose osimertinib; these resolved with erlotinib, although impaired consciousness developed two days after its discontinuation due to thrombocytopenia, followed by rapid clinical decline. (B) Serum tumor markers, including CEA and SLX, showed improvement 1.5 months after the initiation of osimertinib; however, both markers were markedly elevated immediately before the onset of leptomeningeal carcinomatosis. (C) C1–C3 show non‑contrast CT images of the primary lung lesion. Tumor shrinkage was observed after the initiation of osimertinib therapy, and pleural effusion subsequently resolved. C4–C5 show contrast‑enhanced brain MRI. Before treatment initiation, no findings suggestive of metastatic lesions or leptomeningeal carcinomatosis were observed. However, on day 115, contrast enhancement along the cerebral surface and sulci was noted on FLAIR images, suggestive of leptomeningeal carcinomatosis. CEA: carcinoembryonic antigen; SLX: sialyl Lewis X; FLAIR: fluid‑attenuated inversion recovery

On day 29, neutrophils decreased from 1,803/µL (grade 1) to 843/µL (grade 3) without spontaneous recovery. Therefore, the dose was further reduced to 40 mg every other day beginning on August 15 (day 44). She was transferred to another facility for postoperative rehabilitation and discharged home approximately two months later. Imaging before transfer showed shrinkage of the primary lesion, decreased pleural effusion, stable metastatic lesions, and improvement in CEA from 408.9 to 179.1 ng/mL. The course of treatment and observations is shown in Figure 2A.

**Figure 3:**
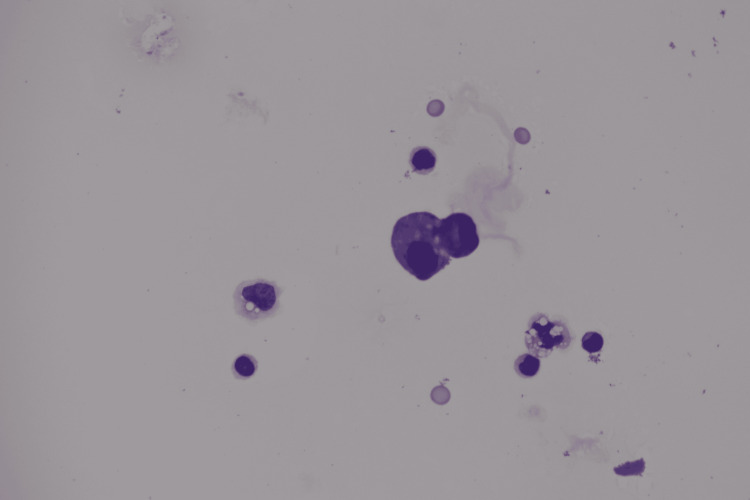
Pathological findings of the cerebrospinal fluid obtained on day 115. Giemsa staining showed a high‑power view demonstrating nuclear irregularity with notching and nuclear polarization.

Shortly after returning home, imaging continued to demonstrate shrinkage of the primary lesion and resolution of pleural effusion (Figure 2C1-2C3), with no enlargement of metastatic sites. However, CEA rose sharply to 946.9 ng/mL, and SLX showed a similar trend (Figure 2B). She presented to the emergency department with dizziness and headache on October 25 (day 115). Contrast‑enhanced brain MRI demonstrated leptomeningeal enhancement along the cerebral sulci on fluid‑attenuated inversion recovery (FLAIR) imaging (Figures 2C4-2C5), and cerebrospinal fluid cytology revealed malignant cells (Figure 3), confirming leptomeningeal carcinomatosis.

Given reports suggesting dose‑dependent efficacy of erlotinib for LMC and the possibility that insufficient cerebrospinal fluid penetration of reduced‑dose osimertinib contributed to disease progression, erlotinib 150 mg/day was initiated as second‑line therapy on October 26 (day 116). As her upper gastrointestinal symptoms had improved, rabeprazole was replaced with famotidine 20 mg/day as a precautionary measure, given the potential association between proton pump inhibitors and thrombocytopenia. Her dizziness and headache resolved; however, on day 120, platelet counts decreased from 236,000/µL to 47,000/µL (grade 3), necessitating treatment interruption (Figure 2). Two days later, she developed impaired consciousness and subsequently died.

## Discussion

Kimura et al. reported that the incidence of LMC in lung adenocarcinoma is approximately 1.5% [3]. Although relatively uncommon, a comparative study demonstrated a significantly higher incidence in patients with EGFR mutations (9.4%) than in those without mutations (1.7%) [4]. LMC is generally known to carry a poor prognosis; before the introduction of EGFR TKIs, survival was reported to be four to six weeks without treatment and two to three months even with therapy [5,6]. In contrast, Li et al. reported a median overall survival of 10 months in patients treated with EGFR TKIs [4], and the phase II BLOSSOM trial evaluating osimertinib for LMC demonstrated a median overall survival of 15 months [7].

In the present case, osimertinib was continued at a reduced dose of 40 mg every other day due to thrombocytopenia. In the AURA3 trial, the objective response rate was 70% with standard‑dose osimertinib, whereas reduced doses of 20 mg/day and 40 mg/day yielded response rates of 50% and 59%, respectively [8]. Consistent with these findings, our patient maintained relatively good control of extracranial disease on reduced‑dose therapy; however, she developed LMC and was considered to have progressive disease. Although no consensus exists regarding second‑line therapy after progression of LMC during EGFR‑TKI treatment, Yajima et al. reported dose‑dependent efficacy of erlotinib in such cases, describing a patient whose neurological symptoms improved on 150 mg/day, recurred after dose reduction to 100 mg/day due to severe skin toxicity, and improved again when the dose was re‑escalated to 150 mg/day [9]. Given that erlotinib has been suggested to have dose‑dependent CNS penetration, full‑dose therapy was considered a reasonable option in our patient. Although cerebrospinal fluid reassessment was not performed, her headache and other symptoms improved markedly, suggesting therapeutic benefit.

Grade 3 or higher thrombocytopenia associated with osimertinib has been reported in 1% of patients in the AURA3 trial [8], and erlotinib‑related thrombocytopenia occurred in 1.8% of patients in the ENSURE trial [10]. Previous reports have described cases in which thrombocytopenia was suspected to be related to concomitant sitagliptin use and improved after its discontinuation, allowing continuation of osimertinib [11], as well as cases in which switching from osimertinib to aumolertinib enabled ongoing treatment [12].

In our case, thrombocytopenia had already occurred after the initiation of osimertinib and prior to the introduction of rabeprazole, so proton-pump inhibitor (PPI)‑associated thrombocytopenia was not strongly suspected. Rabeprazole had been introduced for upper gastrointestinal symptoms, which had improved by the time erlotinib was initiated; therefore, it was replaced with famotidine as a precaution before starting erlotinib. Nevertheless, grade 3 thrombocytopenia developed shortly thereafter, necessitating treatment interruption. Although symptom improvement had been achieved and continuation with platelet transfusion support was considered, the patient developed impaired consciousness only two days after discontinuation, leading to rapid deterioration in performance status and inability to continue oral therapy, ultimately resulting in death. This rapid decline may have reflected the aggressive disease activity present even before initiation of lung cancer treatment.

Dose reduction is an established strategy for managing osimertinib‑induced neutropenia [13]. In our case, although reduced‑dose osimertinib maintained control of extracranial disease, the patient developed LMC. Because the disease progressed rapidly after the onset of LMC, we were unable to evaluate potential EGFR‑TKI resistance mechanisms or measure cerebrospinal fluid drug concentrations; therefore, our interpretation remains speculative. However, given that extracranial disease remained controlled even under reduced dosing and that neurological symptoms improved promptly after switching to erlotinib 150 mg/day, it is possible that dose reduction may have affected CNS disease control. Although based on an animal model, a study evaluating the blood-brain barrier permeability of EGFR‑TKIs in rats reported a free‑brain/free‑plasma concentration ratio (Kpuu) of 0.21 for osimertinib [14], suggesting that cerebrospinal fluid concentrations may also be lower than plasma concentrations in humans.

## Conclusions

This case illustrates that reduced‑dose EGFR TKI therapy, even with a CNS‑penetrant agent such as osimertinib, may be insufficient for preventing or controlling central nervous system involvement, including LMC. Although extracranial disease remained well controlled, the patient developed LMC, and the clinical course raises the possibility that decreased cerebrospinal fluid drug exposure associated with dose reduction could have played a role. Clinicians should be aware that dose modification necessitated by adverse events may affect CNS protection, and careful monitoring for neurological symptoms remains essential in patients receiving reduced‑dose EGFR TKIs.
